# Optimal Corticosteroid Therapy Based on Liver Biopsy for Severe Immune-Mediated Hepatitis During Pembrolizumab Treatment: A Case Report

**DOI:** 10.7759/cureus.104904

**Published:** 2026-03-09

**Authors:** Kotoba Esaki, Hideyuki Horikoshi, Maiko Awashima, Satoshi Nakayama, Yoshiko Kichikawa

**Affiliations:** 1 Department of Internal Medicine, Self-Defense Forces Central Hospital, Tokyo, JPN; 2 Department of Internal Medicine, Mishuku Hospital, Federation of National Public Service Personnel Mutual Aid Associations, Tokyo, JPN; 3 Department of Gastroenterology, Mishuku Hospital, Federation of National Public Service Personnel Mutual Aid Associations, Tokyo, JPN; 4 Department of Respiratory Medicine, Mishuku Hospital, Federation of National Public Service Personnel Mutual Aid Associations, Tokyo, JPN

**Keywords:** corticosteroid therapy, immune-mediated hepatitis, immune-related adverse event, liver biopsy, non-small cell lung cancer, pembrolizumab therapy

## Abstract

Immune checkpoint inhibitors (ICIs), including pembrolizumab, are widely used to treat advanced non-small cell lung cancer; however, they are associated with immune-related adverse events, including immune-mediated hepatitis. Although systemic corticosteroids are recommended as first-line therapy for severe immune-mediated hepatitis, the optimal initial dose remains unclear, and current guidelines specify a wide range of doses. Herein, we report the case of a 76-year-old man with lung adenocarcinoma, a history of resolved hepatitis B virus infection, ulcerative colitis in long-term remission, and latent hereditary spherocytosis who developed grade 3 immune-mediated hepatitis during pembrolizumab monotherapy. Liver biopsy revealed moderate lobular necroinflammation consistent with immune-mediated hepatitis. Based on these histopathological findings, prednisolone at 0.3 mg/kg/day was initiated. Liver enzyme levels improved rapidly, and pembrolizumab was subsequently resumed without recurrence of immune-mediated hepatitis. This case highlights the diagnostic and therapeutic value of liver biopsy in patients with severe immune-mediated hepatitis. Histopathological assessment of inflammatory severity and bile duct involvement may contribute to individualized corticosteroid dosing, promote early recovery, and support safe ICI rechallenge.

## Introduction

Pembrolizumab, a programmed cell death-1 (PD-1) inhibitor, has demonstrated significant survival benefits in patients with advanced non-small cell lung cancer and has become a cornerstone of modern immunotherapy [1]. This monoclonal antibody restores antitumor cytotoxic T-cell activity by reversing tumor- and immune checkpoint-mediated inhibition. However, with its widespread use, immune-related adverse events (irAEs) have emerged as major clinical challenges, reflecting immune dysregulation induced by immune checkpoint blockade [2]. Among these irAEs, immune-mediated hepatitis occurs in approximately 5-10% of patients receiving immune checkpoint inhibitor (ICI) monotherapy. Severe hepatitis, which often presents with asymptomatic elevations in serum transaminases requiring ICI discontinuation, has been reported in fewer than 5% of cases (grade ≥3 according to the Common Terminology Criteria for Adverse Events (CTCAE) severity classification) [2-4].

Severe immune-mediated hepatitis frequently necessitates treatment interruption or permanent discontinuation of ICIs and is associated with unfavorable clinical outcomes [5,6]. A recent systematic review and meta-analysis reported that approximately 16% of cases are refractory to corticosteroid therapy and that 22% of patients experience recurrence of hepatitis after ICI rechallenge [7]. These findings underscore the clinical importance of optimizing diagnostic strategies and therapeutic decision-making for immune-mediated hepatitis.

International guidelines recommend multidisciplinary consultation with a hepatologist and consideration of liver biopsy in cases of severe immune-mediated hepatitis or refractory hepatotoxicity [5,6,8]. Liver biopsy can help differentiate immune-mediated hepatitis from other causes of liver injury, including autoimmune hepatitis, drug-induced liver injury, and immune-related cholangitis, and can also assess the severity and histologic pattern of inflammation [8-10]. Histological features, such as the degree of necroinflammation and the presence of bile duct injury, have been suggested to correlate with steroid responsiveness and clinical outcomes [11,12]. Nevertheless, liver biopsy is not routinely performed in clinical practice because of its invasiveness, and treatment decisions are often based solely on biochemical and imaging findings. This limitation may hinder the accurate assessment of histologic severity and create uncertainty when determining the optimal initial corticosteroid dose.

Guidelines recommend systemic corticosteroids as first-line therapy for grade 3 or higher immune-mediated hepatitis; however, the recommended initial dose varies substantially, ranging from 1 to 2 mg/kg/day [5,6,13]. These discrepancies likely reflect the limited high-quality evidence available and differences in clinical emphasis on balancing hepatic toxicity control against the risks of excessive immunosuppression. Such variability in guideline recommendations may influence clinical decision-making regarding the initial corticosteroid dose in individual patients. Excessive corticosteroid exposure may increase the risk of infectious complications and delay resumption of anticancer therapy, whereas the safety and efficacy of lower-dose corticosteroid strategies remain insufficiently established [14]. These uncertainties highlight the need for objective tools to guide therapeutic decision-making in severe immune-mediated hepatitis.

Herein, we report a case of lung adenocarcinoma in which grade 3 immune-mediated hepatitis developed during pembrolizumab monotherapy. Liver biopsy confirmed the diagnosis and revealed moderate necroinflammatory changes without bile duct injury. Guided by these histopathological findings, treatment with 0.3 mg/kg/day of corticosteroids was initiated, resulting in early recovery and safe ICI rechallenge.

## Case presentation

A 76-year-old man was admitted for a liver biopsy with no specific complaints. His medical history included cholecystitis, resolved hepatitis B virus infection (HBsAg-negative, anti-HBc-positive, anti-HBs-positive, and undetectable HBV-DNA), ulcerative colitis in 10-year remission, hypertension, latent hereditary spherocytosis, left renal cell carcinoma, and allergic rhinitis. The patient had no significant family history. He had a 22.5 pack-year smoking history and a long history of alcohol consumption (approximately 70 g/day for 15 years). He had no known drug or food allergies. His regular medications at admission included mesalazine (2,400 mg), *Clostridium butyricum *MIYAIRI 588, and amlodipine besylate (2.5 mg), with no recent changes or newly introduced medications.

Two years before the present admission, in late March, a follow-up computed tomography scan performed after surgery for left renal cell carcinoma (with no recurrence) revealed a nodular lesion with ground-glass opacity in the left lingular segment. The patient was referred to the Department of Respiratory Medicine for further evaluation. Direct invasion into the left lower lobe was suspected, and in May of the same year, he underwent a left upper lobectomy with combined resection of segment 6 of the left lower lobe. Histopathological examination revealed invasive lung adenocarcinoma, staged as pT4N0M0, stage IIIA, according to the eighth edition of the Union for International Cancer Control classification. Genetic analysis using the Oncomine CDx five assay detected no mutations in *EGFR*, *BRAF*, *ALK*, *ROS1*, or *RET*. The programmed death-ligand 1 (PD-L1) tumor proportion score, assessed by the 22C3 pharmDx assay, was 1-10%. He subsequently received four cycles of postoperative adjuvant chemotherapy with carboplatin plus nab-paclitaxel.

In May, one year before the present admission, postoperative recurrence was detected. He received four cycles of carboplatin plus pemetrexed as first-line treatment for recurrent advanced lung adenocarcinoma. Immediately after the first cycle of pemetrexed maintenance therapy, left eyelid edema developed. Therefore, beginning in September of the same year, he received four cycles of carboplatin plus paclitaxel as second-line treatment; however, radiologic evaluation demonstrated disease progression. Given the PD-L1 tumor proportion score of 1-10%, pembrolizumab monotherapy was initiated as third-line treatment. At treatment initiation, liver function tests were normal, and imaging studies revealed no findings suggestive of liver metastasis or biliary disease. For clarity, day 1 was defined as the first day of pembrolizumab administration.

On day 22, the first blood test after starting pembrolizumab revealed elevated aspartate aminotransferase (AST) and alanine aminotransferase (ALT) levels of 105 U/L and 89 U/L, respectively, prompting referral to a hepatologist.

On physical examination at admission, his height was 169 cm, and his weight was 74 kg. He was afebrile, and his blood pressure and heart rate were within normal ranges. Respiratory rate was 24 breaths per minute, and peripheral oxygen saturation on room air was 97%. He reported an exertional cough. No scleral icterus was observed, although conjunctival pallor was noted. Cardiac and pulmonary auscultations were unremarkable. The abdomen was flat and soft, without tenderness, and no skin rash was observed on the trunk or extremities. His Eastern Cooperative Oncology Group performance status was 1.

Laboratory findings at onset demonstrated AST and ALT elevation with hyperbilirubinemia. Anemia with elevated reticulocyte count, indirect hyperbilirubinemia, elevated lactate dehydrogenase levels, and decreased haptoglobin suggested hemolytic anemia. A direct antiglobulin test was negative, and spherocytosis was observed on the peripheral smear. These findings were compatible with hereditary spherocytosis rather than newly developed autoimmune hemolytic anemia. Accordingly, mild hyperbilirubinemia had been present at baseline, ranging from 0.9 to 2.0 mg/dL. Serological testing was negative for hepatitis B virus DNA, hepatitis C virus antibody, cytomegalovirus antigenemia, and Epstein-Barr virus. Antinuclear antibody was negative, and serum immunoglobulin levels were normal, suggesting viral hepatitis and autoimmune liver diseases, including autoimmune hepatitis and primary biliary cholangitis, were unlikely. Mesalazine-induced liver injury was also considered in the differential diagnosis; however, the patient had been receiving a stable dose for more than 10 years without prior hepatic abnormalities, and therefore mesalazine-induced liver injury was considered unlikely, and the medication was continued.

**Figure 1:**
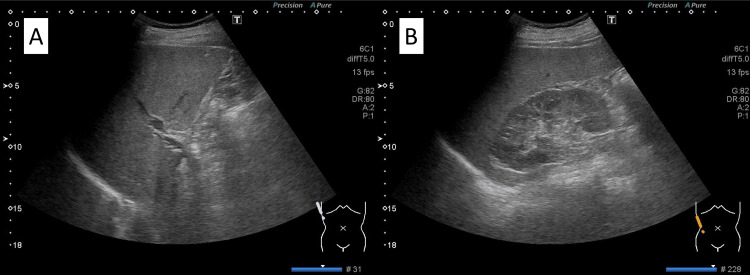
Abdominal ultrasonography of the liver Ultrasound examination of the abdomen showed increased echogenicity with a blunt liver edge. The gallbladder is absent, consistent with prior cholecystectomy.

**Figure 2:**
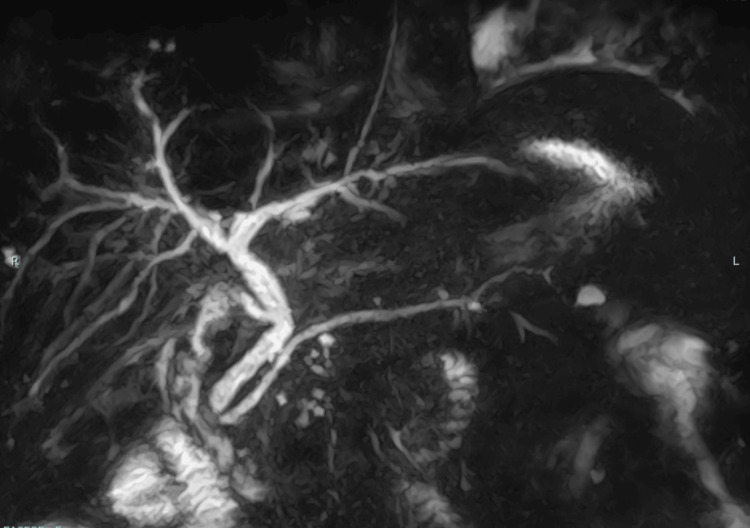
MRCP of the biliary tract MRCP showed mild extrahepatic bile duct dilation without evidence of biliary stricture, wall thickening, or intrahepatic bile duct dilation. MRCP, magnetic resonance cholangiopancreatography

To evaluate possible hepatobiliary disease, abdominal ultrasonography (Figure 1) and magnetic resonance cholangiopancreatography (MRCP; Figure 2) were performed. Abdominal ultrasonography revealed a blunt liver edge with a smooth surface and slightly increased echogenicity, consistent with mild fatty liver disease. Splenomegaly with a longitudinal diameter of 137 mm was also noted. The pancreas was visualized without evidence of enlargement, parenchymal heterogeneity, or peripancreatic fluid collection. On MRCP, mild dilation of the extrahepatic bile duct was observed, likely related to prior cholecystectomy; however, no biliary stricture, wall thickening, or intrahepatic bile duct dilation was identified.

**Figure 3:**
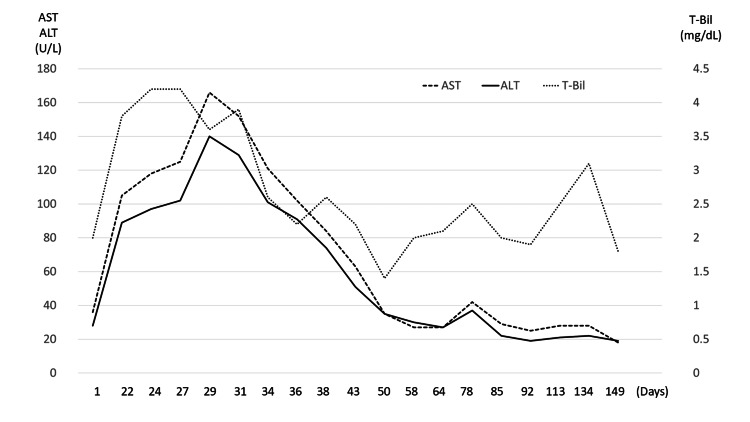
Serial changes in serum AST, ALT, and T-Bil during the clinical course Serial changes in serum AST (dashed line), ALT (solid line), and T-Bil (dotted line) over time are shown. AST and ALT levels are plotted on the left y-axis (U/L), and T-Bil is plotted on the right y-axis (mg/dL). T-Bil remained mildly elevated because of underlying chronic hemolysis associated with hereditary spherocytosis (baseline range: 0.9-2.0 mg/dL). ALT, alanine aminotransferase; AST, aspartate aminotransferase; T-Bil, total bilirubin

**Figure 4:**
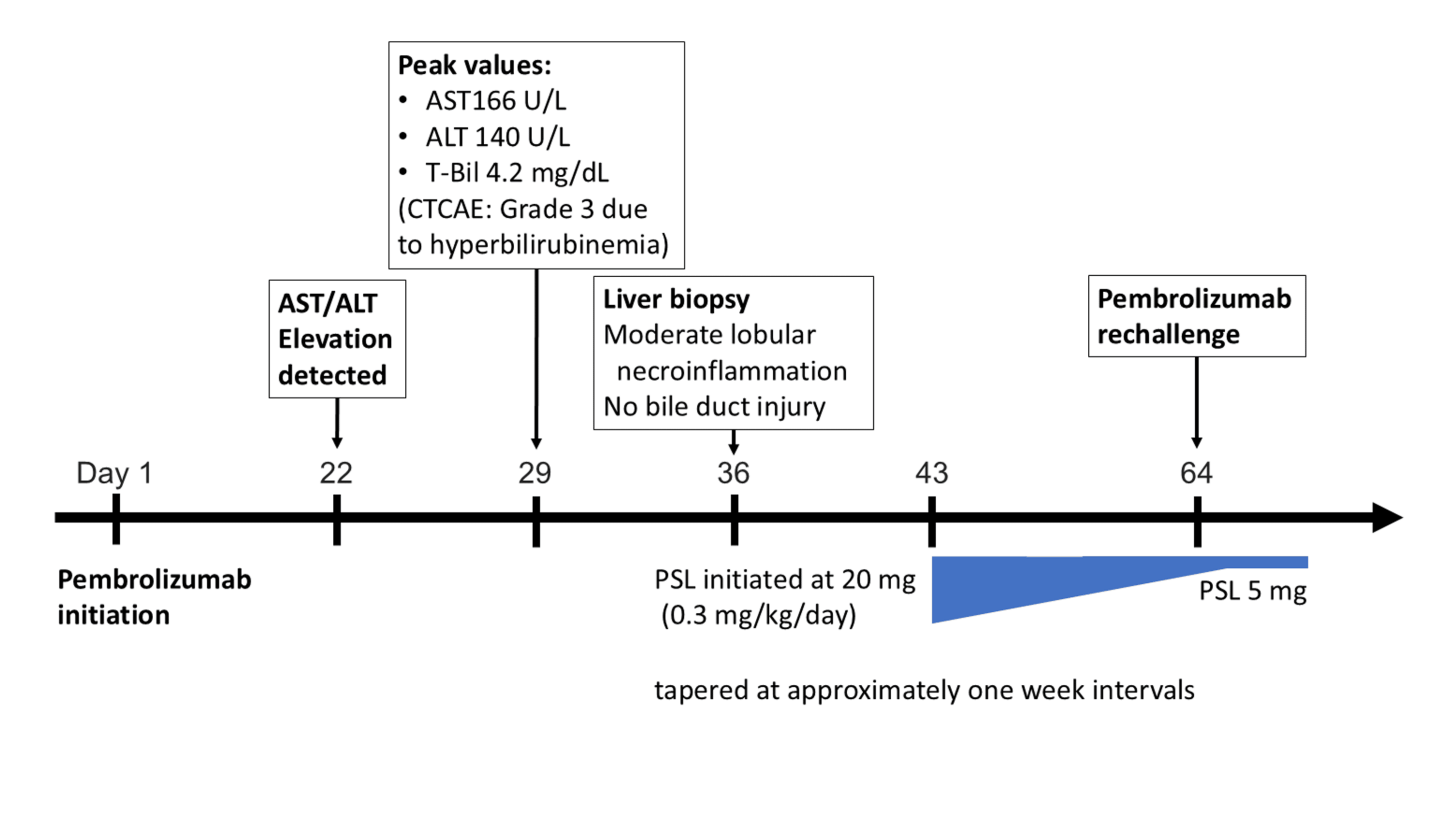
Clinical timeline of immune-mediated hepatitis and treatment course Timeline summarizing key clinical events, including onset of liver enzyme elevation, imaging studies, liver biopsy, initiation and tapering of prednisolone, and pembrolizumab rechallenge. ALT, alanine aminotransferase; AST, aspartate aminotransferase; CTCAE, Common Terminology Criteria for Adverse Events; PSL, prednisolone; T-Bil, total bilirubin

Regarding the clinical course (Figure 3, Figure 4), on day 29, laboratory evaluation revealed grade 2 AST elevation, grade 2 ALT elevation, grade 3 hyperbilirubinemia, grade 3 hyperamylasemia, and grade 3 anemia according to the CTCAE version 5.0 [4]. Despite hyperamylasemia (350 IU/L) and elevated serum lipase (301 U/L), the patient reported no abdominal symptoms, including typical epigastric pain. Abdominal ultrasonography showed no pancreatic enlargement or peripancreatic abnormalities, and the pancreatic enzyme levels showed a gradual decline without specific treatment. These findings did not fulfill the diagnostic criteria for acute pancreatitis, and clinically significant ICI-related pancreatitis was not diagnosed. Immune-mediated hepatitis associated with pembrolizumab was suspected, and a liver biopsy was performed from segment 6 of the right hepatic lobe using a 16-gauge core needle on day 36.

**Figure 5:**
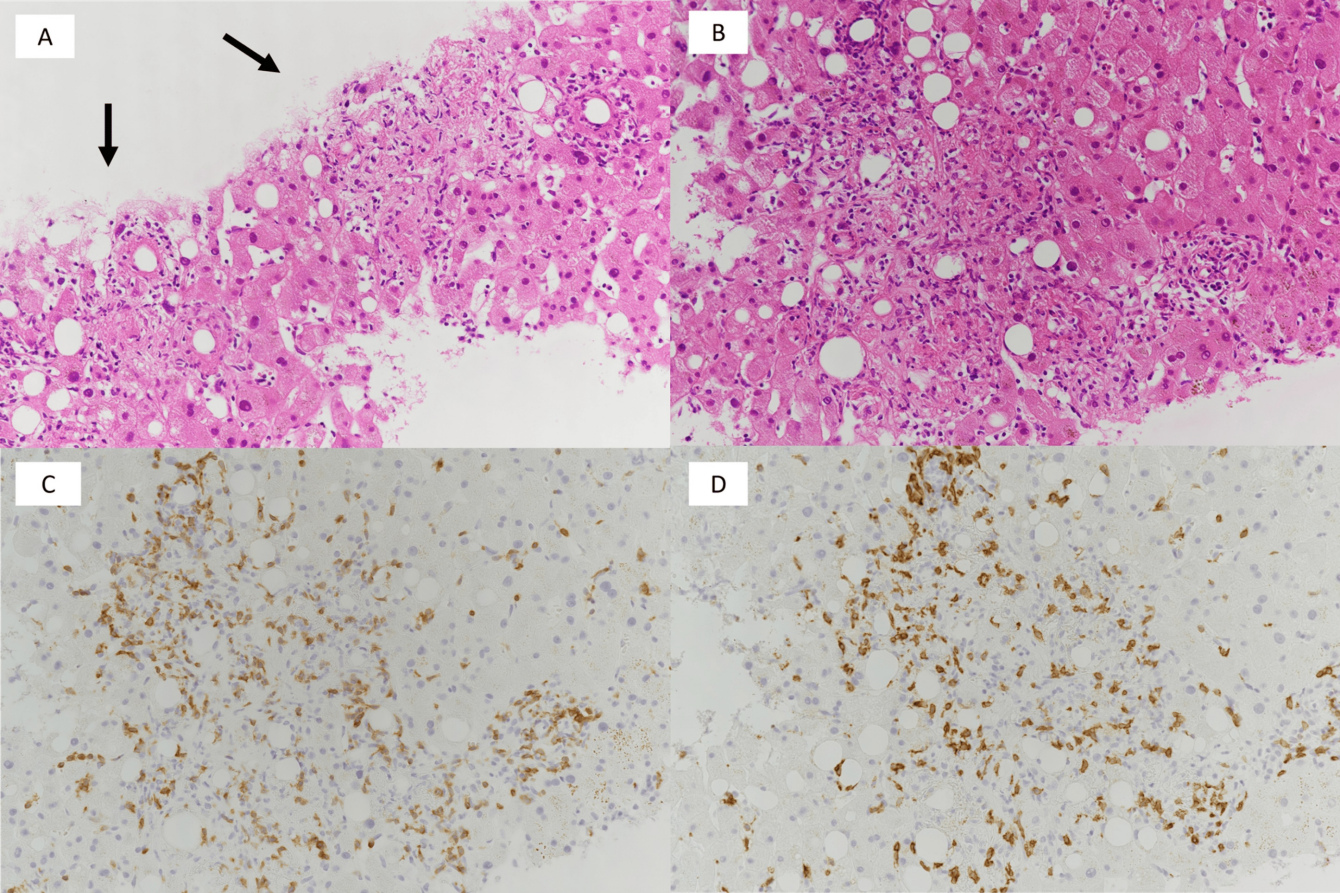
Histopathological findings of the liver biopsy (A) H&E staining (×200) showing epithelioid granulomas in the hepatic lobule and periportal area (arrow). (B) H&E staining (×200) showing granulomas composed predominantly of lymphocytic inflammatory infiltrates with neutrophils, along with mild macrovesicular steatosis. (C) Immunohistochemical staining for CD3 (×200) showing predominant infiltration of CD3-positive T lymphocytes in the corresponding area. (D) Immunohistochemical staining for CD8 (×200) showing predominant infiltration of CD8-positive T lymphocytes in the corresponding area.

Histopathological examination revealed inflammatory changes predominantly characterized by lymphocytic infiltration within the hepatic lobules. Immunohistochemical analysis showed a predominance of CD3-positive T lymphocytes, whereas CD20-positive B lymphocytes were scarce. Granuloma formation was prominent, and CD8-positive lymphocytes were slightly predominant. These findings were consistent with hepatic injury associated with ICI therapy (Figure 5). The liver biopsy revealed only minimal steatosis, involving <5% of hepatocytes.

Based on the diagnosis of irAEs due to lymphocyte activation following ICI therapy, treatment was initiated on day 43 in accordance with the treatment flowchart in Ito et al.’s study [10]. Ursodeoxycholic acid (600 mg/day in three divided doses) and oral prednisolone (0.3 mg/kg/day) were administered. After discussion with hepatology specialists, corticosteroid therapy was initiated at a lower dose than that recommended by the Japanese Society of Medical Oncology (JSMO) guidelines [13], as an individualized therapeutic approach, considering that liver biopsy demonstrated moderate hepatic inflammation. Elevated hepatobiliary enzyme levels, serum amylase levels, and anemia gradually improved, and liver enzyme levels normalized by day 50. Prednisolone was subsequently tapered by 5 mg at approximately one-week intervals with close monitoring of liver function tests, considering the favorable biochemical response and the need for early resumption of anticancer therapy. When the dose was tapered to 5 mg/day, pembrolizumab was resumed on day 64. The dose was further tapered to 5 mg/day on day 67 and maintained thereafter. Four additional cycles of pembrolizumab were then administered at three- to four-week intervals without recurrence of immune-mediated hepatitis.

## Discussion

We report a case of lung adenocarcinoma in which liver biopsy enabled a definitive diagnosis of immune-mediated hepatitis and informed an individualized corticosteroid strategy, leading to early fulfillment of the criteria for ICI rechallenge.

In this case, the differential diagnosis of liver injury initially included hepatitis B virus reactivation, metabolic dysfunction-associated alcohol-related liver disease, mesalazine-induced liver injury, autoimmune hepatitis, and immune-related cholangitis, in addition to immune-mediated hepatitis, based on the patient’s clinical background and laboratory findings. Viral hepatitis was excluded based on serological testing; however, the etiology of liver injury remained uncertain. Imaging studies ruled out metastatic liver tumors and vascular thrombosis but demonstrated underlying hepatic steatosis. Mesalazine-induced liver injury was considered unlikely, given the long-term unchanged administration for more than 10 years, absence of prior hepatic abnormalities, and lack of a temporal relationship between drug exposure and liver enzyme elevation. Differentiation among immune-related cholangitis, autoimmune hepatitis, and immune-mediated hepatitis remained difficult based on biochemical and radiologic findings alone, as previously reported [11]. A liver biopsy allowed pathological exclusion of alternative etiologies other than immune-mediated hepatitis and accurate assessment of the severity and histologic pattern of hepatic inflammation [8,10]. This enabled a definitive diagnosis of immune-mediated hepatitis and provided essential information for therapeutic decision-making.

Although liver biopsy is not routinely performed in clinical practice because of its invasiveness, recent studies have demonstrated its clinical utility in managing immune-mediated hepatitis. Riveiro-Barciela et al. reported that an algorithm incorporating liver biopsy findings allowed corticosteroid therapy to be avoided in approximately two-thirds of patients with severe ICI-induced liver injury [9]. While that study focused primarily on steroid avoidance rather than dose reduction, it underscores the broader principle that histopathological evaluation may refine treatment strategies. Based on the findings of that study, steroid avoidance could theoretically have been considered. After consultation with hepatology specialists, we aimed to pursue early ICI resumption consistent with the JSMO guidelines and therefore decided to initiate corticosteroid therapy. Furthermore, retrospective studies have suggested that histologic patterns characterized by predominant lobular inflammation without bile duct injury may be associated with better steroid responsiveness, whereas bile duct-dominant patterns are more frequently steroid refractory [11,12]. These associations are primarily derived from small retrospective cohorts and require validation in larger prospective cohorts. However, they suggest that liver biopsy provides objective pathological information not obtainable from biochemical testing alone and may support individualized treatment strategies.

Current international guidelines recommend systemic corticosteroids as first-line therapy for grade 3 or higher immune-mediated hepatitis; however, the recommended initial dose varies substantially, ranging from 1 to 2 mg/kg/day [5,6,13]. High-dose corticosteroid therapy may increase the risk of infectious complications and delay the resumption of anticancer treatment, whereas the safety and efficacy of lower-dose strategies have not been fully established [14]. Thus, objective indicators to guide corticosteroid dosing in patients with severe immune-mediated hepatitis remain an important yet unmet clinical need.

In this case, a liver biopsy demonstrated moderate lobular necroinflammation without bile duct injury, a pathological pattern associated with favorable steroid responsiveness [11,12]. Although these observations are derived primarily from small retrospective studies, the patient’s overall prognosis in this case was considered to be determined mainly by the underlying adenocarcinoma rather than hepatitis. In this clinical context, hepatology and respiratory specialists jointly discussed the management strategy. Prednisolone was initiated at 0.3 mg/kg/day, a dose lower than that recommended in current guidelines for grade 3 immune-related hepatitis, with the aim of facilitating early ICI rechallenge. This approach was individualized to this patient and was not intended as a general recommendation. Liver enzyme levels improved rapidly, enabling early fulfillment of the criteria for ICI rechallenge.

From a pathophysiological perspective, immune-mediated hepatitis is thought to result from excessive activation of cytotoxic T lymphocytes induced by PD-1 blockade, leading to immune-mediated hepatocellular injury [2,3]. Corticosteroids suppress T-cell activation and pro-inflammatory cytokine production, thereby mitigating hepatic inflammation. Previous studies suggest that cases characterized by predominant lobular inflammation without bile duct injury respond well to corticosteroids, whereas those with cholangitic or bile duct-dominant patterns are frequently steroid-refractory [11,12]. Moreover, histologic evidence of bile duct injury has been associated with a significantly higher risk of recurrence after ICI rechallenge [12]. A recent meta-analysis reported recurrence of immune-mediated hepatitis in approximately 22% of patients after ICI rechallenge [7]. Together, these observations provide a pathological rationale for stratifying therapeutic intensity based on histologic patterns.

In this patient, therapeutic options for adenocarcinoma were limited, making early resumption of anticancer therapy clinically important for preventing tumor progression. International guidelines generally discourage ICI rechallenge after grade 3 or higher hepatic toxicity [5,6]. In contrast, the JSMO guidelines allow ICI rechallenge under specific conditions, including recovery of liver function to baseline or grade 1 level, tapering of corticosteroids to ≤10 mg/day over at least four to six weeks, and absence of fulminant hepatitis or severe cholangitis [13]. In the present case, prednisolone was tapered in a stepwise fashion at intervals of one week based on biochemical response. Given the favorable biochemical response and the rapid normalization of liver enzyme levels, corticosteroids were tapered relatively early under close monitoring after consultation with hepatology specialists, taking into account both the potential steroid-related toxicity and the need for early resumption of ICI therapy. Consequently, these criteria were satisfied, and ICI rechallenge was successfully performed without recurrence of immune-mediated hepatitis.

Previous studies have shown that patients with histologic evidence of bile duct injury during the initial episode of immune-mediated hepatitis have a significantly higher risk of recurrence after ICI rechallenge [12]. In this case, the absence of bile duct injury and the presence of only moderate necroinflammatory changes on liver biopsy may have contributed to the favorable clinical course observed in this patient, although causality cannot be established from a single case.

Overall, these findings suggest that histopathological evaluation may provide important insights into steroid responsiveness and the risk of recurrence in immune-mediated hepatitis. Nevertheless, prospective studies are required to determine whether specific histopathological patterns, including hepatocellular (lobular) inflammation without bile duct injury, can reliably guide corticosteroid dosing or predict safe ICI rechallenge. As this is a single case report, the generalizability of these findings remains limited.

## Conclusions

In patients with immune-mediated hepatitis associated with ICIs, liver biopsy may provide valuable information not only for differential diagnosis but also for assessing histologic severity and patterns of inflammation. In this case, a pathology-guided approach and multidisciplinary decision-making promoted earlier recovery and supported safe ICI rechallenge. Although additional case accumulation is required, these findings suggest that liver biopsy may play an important role in individualizing corticosteroid dosing and optimizing therapeutic strategies for severe immune-mediated hepatitis.
